# Comparison of clinical outcomes between early and delayed allogeneic hematopoietic stem cell transplantation for severe aplastic anemia: a single-center retrospective study

**DOI:** 10.1007/s00277-026-06869-6

**Published:** 2026-03-18

**Authors:** Zhengwei Tan, Yuechao Zhao, Huijin Hu, Qinghong Yu, Yu Zhang, Tonglin Hu, Dijiong Wu, Baodong Ye, Wenbin Liu

**Affiliations:** 1 Department of Hematology, The First Affiliated Hospital of Zhejiang, Chinese Medical University (Zhejiang Provincial Hospital of Traditional Chinese Medicine), Hangzhou, China; 2 The First School of Clinical Medicine, Zhejiang Chinese Medical University, Hangzhou, China

**Keywords:** Aplastic anemia, Hematopoietic stem cell transplantation, Graft-versus-host disease, Waiting time, Overall survival

## Abstract

Allogeneic hematopoietic stem-cell transplantation (allo-HSCT) is the only curative therapy for severe aplastic anemia (SAA), yet the prognostic impact of the diagnostic-to-transplant interval on first allo-HSCT remains contentious. To determine whether a waiting time > 1 year from diagnosis to first allo-HSCT compromises engraftment, virus reactivation, GVHD, OS and GRFS in SAA. A single-center retrospective cohort study of 255 consecutive SAA patients receiving their first allo-HSCT between 2018 and 2025. After 1:2 propensity-score matching (caliiper 0.2), patients were stratified into Early (≤ 1 year, *n* = 170) and Delayed (> 1 year, *n* = 85) groups. Baseline characteristics were well balanced. The Early group exhibited a significantly lower CMV reactivation rate (24.1% vs. 40.0%, *P* = 0.008). No significant differences were observed in grade II-IV aGVHD, cGVHD, 5-year OS or GRFS. Subgroup analyses demonstrated superior survival among patients aged ≤ 40 years, those with MSD donors and received FABT-based regimen. Early allo-HSCT improves transplant outcomes in SAA by reducing CMV reactivation, especially in very severe cases. Eligible patients should be referred promptly and transplanted without delay.

## Introduction

Severe aplastic anemia (SAA) is an acquired hematologic disorder characterized by bone marrow hematopoietic failure, primarily driven by T lymphocyte-mediated immune destruction of hematopoietic stem and progenitor cells [1]. Without effective treatment, patients often succumb early to severe complications such as infections and hemorrhage, with a two-year mortality rate reaching up to 50% after diagnosis [2]. Allogeneic hematopoietic stem cell transplantation (allo-HSCT) currently represents the only curative treatment for SAA. Particularly in younger patients with HLA-matched sibling donors (MSD), long-term overall survival (OS) rates can reach 80%~90% [3, 4]. For those lacking a MSD, the refinement of haploidentical (HID) and matched unrelated donor (MUD) transplantation has significantly expanded donor availability, substantially improving access to allo-HSCT [5, 6]. Furthermore, the widespread adoption of fludarabine-based reduced-intensity conditioning regimens combined with post-transplant cyclophosphamide (PTCy) has further enhanced transplant safety and reduced the incidence of graft-versus-host disease (GVHD) [7].

Despite continuous advancements in transplantation techniques, the optimal timing for transplantation after diagnosis in patients with SAA and its impact on prognosis remain subjects of significant debate. Historically, guidelines recommended age and donor type-stratified approaches, with IST as the primary option for patients lacking a MSD [8]. However, studies have found that delayed transplantation leads to a prolonged need for blood product transfusions, which not only increases the risk of viral transmission (e.g., CMV and EBV) through blood products but may also induce the production of donor-specific antibodies (DSA), thereby affecting engraftment rates [9–11]. In contrast, early transplantation can avoid the risks of infection and bleeding associated with prolonged cytopenia, reduce iron overload and graft rejection caused by repeated transfusions, and prevent treatment delays due to IST failure. One study found that an interval of > 6 months from diagnosis to transplantation in aplastic anemia patients is a significant predictor of mortality, and those with a delay of over 6 months had higher transplant failure rates [12]. Meanwhile, another study indicated that when using a PTCy-based GVHD prophylaxis regimen, delayed transplantation did not increase the incidence of grade II–IV aGVHD or cGVHD [13]. These conflicting results underscore the necessity of re-evaluating the clinical significance of transplantation timing, particularly by controlling for key confounding variables such as donor type, conditioning regimen, patient age, and disease severity.

To clarify the independent prognostic value of transplant timing, we employed a propensity score matching approach to compare the effects of early transplantation (≤ 1 year) versus delayed transplantation (> 1 year) on transplant outcomes, while controlling for confounding factors such as donor type, conditioning regimen, HCT-CI score, and DSA status. Given that the interval from diagnosis to transplantation is generally prolonged in Chinese patients with SAA due to factors such as donor screening, infection control, and economic constraints, defining the acceptable range of this time window holds significant practical implications for optimizing treatment strategies and rationally allocating medical resources. This study aims to provide high-quality evidence for determining the optimal transplant timing in SAA patients and to propose targeted intervention strategies for risk-stratified management of those undergoing delayed transplantation.

## Patients and methods

### Patients

This study retrospectively enrolled all severe aplastic anemia (SAA) and very severe aplastic anemia (VSAA), who underwent first allogeneic hematopoietic stem cell transplantation (allo-HSCT) at our center between January 2018 and January 2025. The diagnosis and classification of AA adhered to the Camitta criteria [14]. Based on the interval from initial diagnosis to transplantation, patients were categorized into Early group (≤ 1 year) and a Delayed group (> 1 year). To control for potential confounders, propensity score matching was performed in a 1:2 ratio with a caliper value of 0.2. Ultimately, 255 AA patients were included, comprising 170 in the Early group and 85 in the Delayed group. The inclusion criteria were as follows: 1). confirmed diagnosis of SAA or VSAA; 2). underwent first allo-HSCT; 3). availability of complete clinical data.

## Transplantation procedures

Donor sources comprised HLA-matched sibling donors, haploidentical donors, and matched unrelated donors. The conditioning regimen was consistent with our previously study [15] and included three regimens: FCA, FABT, and Bucy. Specific dosing schedules were as follows: FCA regimen: Fludarabine (Fu) 30 mg/m^2^/day from day − 9 to -5; cyclophosphamide (CTX) 20 ~ 40 mg/kg/day from day − 5 to -2; and ATG (Sanofi, rabbit source) 2.5 ~ 3.5 mg/kg/day from day − 5 to -2. FABT regimen: Fu 30 mg/m^2^/day from day − 7 to -3; ATG (Sanofi, rabbit source) 2 mg/kg/day from day − 7 to -5; busulfan (Bu) 3.2 mg/kg on day − 4; -3d to -2d, CTX 25 mg/kg/day from day − 3 to -2; and thiotepa (TT) 5 mg/kg on day − 2. Bucy regimen: Bu 3.2 mg/kg/day from day − 7 to -6; CTX 50 mg/kg/day from day − 5 to -2; and ATG (Sanofi, rabbit source) 2.5 mg/kg/day from day − 5 to -2.

The primary difference between groups was that the FABT group received high-dose CTX (40 mg/kg/day from day + 3 to + 4), whereas the FCA and Bucy cohorts received a short course of methotrexate (MTX 15 mg/m^2^ on day + 1 and 10 mg/m^2^ on days + 3, +6, and + 11). All three groups received cyclosporine A (CsA) or tacrolimus (FK506) and mycophenolate mofetil (MMF) for GVHD prophylaxis.

On day − 1, mesenchymal stem cells (MSCs) and/or umbilical cord blood stem cells (UCBs) are infused. MSCs were provided by the Shandong Provincial Cord Blood Bank at a dose of 1 × 10⁶/kg. MSCs and UCBs were infused 24 h prior to donor stem cell transfusion and used only once.

## Definition and assessment

Neutrophil (NE) Engraftment: An absolute NE count greater than 0.5 × 10⁹/L for three consecutive days. Platelet (PLT) Engraftment: A PLT count greater than 20 × 10⁹/L for seven consecutive days without transfusion support. Acute GVHD (aGVHD) and Chronic GVHD (cGVHD): aGVHD diagnosis was based on the modified Glucksberg criteria [16], and cGVHD diagnosis on the 2014 revised NIH consensus criteria [17]. Overall Survival (OS): Defined as the time from HSCT to death or the date of last follow-up. GVHD Relapse-Free Survival (GRFS): The time from HSCT to the occurrence of grade III-IV aGVHD, extensive cGVHD, relapse, or death. CMV reactivation is defined as CMV-DNA copy number exceeding 10³ copies/mL in plasma sample on two consecutive tests. EBV reactivation is defined as EBV-DNA copy number exceeding 10⁴ copies/mL in whole blood sample on two consecutive tests. Primary graft failure (PGF), Secondary graft failure (SGF) and Efficacy Grading follows the Criteria [18]. Efficacy Grading includes Complete Response (CR), Partial Response (PR), and No Response (NR), where the Overall Response (OR) = CR + PR.

### Statistical analysis

Statistical analyses were conducted using SPSS version 27.0 (IBM, Armonk, NY) and GraphPad Prism version 8.0 (GraphPad Software, San Diego, CA). Continuous variables that were not normally distributed are presented as median (range) and were compared using the Mann-Whitney U test. Categorical variables are expressed as frequencies and percentages and compared using the chi-square test. Survival analysis was performed using the Kaplan-Meier method, and survival curves were compared using the log-rank test. A *P*-value of less than 0.05 was considered statistically significant.

## Results

### Patient characteristics

Using propensity-score matching, 255 eligible patients were ultimately enrolled, comprising 170 in the Early group and 85 in the Delayed group. No statistically significant differences in baseline characteristics were observed between the two cohorts. Among 255 enrolled patients, ANC distribution at diagnosis was as follows: ANC < 200/µl (VSAA): 78 (30.6%); ANC 200 ~ 500/µl (typical SAA): 91 (35.7%); and ANC > 500/µl (meeting reticulocyte and platelet count criteria): 86 (33.7%). Among 170 patients in the Early-HSCT group, 166 did not receive IST within 1 year of diagnosis due to critical illness, donor screening, or other factors. Twenty-one (12.7%) died while awaiting transplantation; the primary causes of death were severe infection (3.0%) and fatal hemorrhage (4.8%), with the remaining 8 cases attributable to multi-organ failure. Detailed baseline data are presented in Table 1.

**Table 1:** Baseline data of two groups

Characteristic	Early-HSCT Group	Delayed-HSCT Group	*P* value
Number	170	85	
Sex, n ( % )			0.860
Male	89 ( 52.4 )	43 ( 50.6 )	
Female	81 ( 47.6 )	42 ( 49.4 )	
Age, yr, median ( range )	31 ( 7 ~ 66 )	31 ( 8 ~ 60 )	0.256
Donor sex, n ( % )			0.526
Male	107 ( 62.9 )	50 ( 58.8 )	
Female	63 ( 37.1 )	35 ( 41.2 )	
Donor, Age, yr, median ( range )	28 ( 5 ~ 55 )	28 ( 7 ~ 53 )	0.488
Interval from Diagnosis to HSCT, month, median ( range )	4 ( 1 ~ 12 )	84 ( 13 ~ 180 )	**0.001**
Blood values before HSCT, median ( range )			
WBC	1.7 ( 0 ~ 2.9 )	1.7 ( 0.1 ~ 2.9 )	0.331
ANC	0.4 ( 0 ~ 1.8 )	0.4 ( 0 ~ 1.9 )	0.157
HGB	57 ( 27 ~ 69 )	57 ( 14 ~ 68 )	0.240
PLT	12 ( 1 ~ 27 )	12 ( 1 ~ 28 )	0.694
Ferritin	789 ( 7 ~ 11922 )	793 ( 32 ~ 17747 )	0.130
Disease classification, n ( % )			0.069
SAA	110 ( 64.7 )	67 ( 78.8 )	
VSAA	60 ( 35.3 )	18 ( 21.2 )	
HCT-CI, n ( % )			0.429
0	126 ( 74.1 )	59 ( 69.4 )	
≥ 1	44 ( 25.9 )	26 ( 30.6 )	
DSA, n ( % )			0.488
Negative	138 ( 81.2 )	72 ( 84.7 )	
Positive	32 ( 18.8 )	13 ( 15.3 )	
ABO mismatch, n ( % )			0.096
Match	84 ( 49.4 )	52 ( 61.2 )	
Mismatch	86 ( 50.6 )	33 ( 38.8 )	
HLA, n ( % )			0.152
MSD	23 ( 13.5 )	10 ( 11.8 )	
HID	127 ( 74.7 )	58 ( 68.2 )	
MUD	20 ( 11.8 )	17 ( 20.0 )	
IST, n ( % )	4 ( 2.4 )	5 ( 5.9 )	0.151
PNH, n ( % )	10 ( 5.9 )	8 ( 9.4 )	0.301
Conditioning regimen, n ( % )			0.094
FABT	43 ( 25.3 )	12 ( 14.1 )	
FCA	107 ( 62.9 )	53 ( 62.4 )	
Bucy	20 ( 11.8 )	20 ( 23.5 )	
Graft source, n ( % )			0.140
PBSCs	34 ( 20 )	24 ( 28.2 )	
BMSCs + PBSCs	136 ( 80 )	61 ( 71.8 )	
Umbilical cord blood, n ( % )	67 ( 39.4 )	41 ( 48.2 )	0.180
Mesenchymal stem cell, n ( % )	77 ( 45.3 )	37 ( 43.5 )	0.790
CD34 + cell count,×10^6^/kg, median ( range )	6.01 ( 1.95 ~ 20.38 )	5.99 ( 1.69 ~ 16.84 )	0.193
Letermovir, n ( % )	38 ( 22.4 )	18 ( 21.2 )	0.831

Significant *P* values are in bold type

WBC: White Blood Cell, ANC: Absolute Neutrophil Count, HGB: Hemoglobin, PLT: Platelet, SAA: Severe Aplastic Anemia, VSAA: Very Severe Aplastic Anemia, HCT-CI: Hematopoietic Cell Transplantation-Comorbidity Index, DSA: Donor Specific Antibody, MSD: Matched Sibling Donor, HID: Haploidentical Donor, MUD: Matched Unrelated Donor, IST: Immunosuppressive Therapy, PNH: Paroxysmal Nocturnal Hemoglobinuria, PBSCs: Peripheral Blood Stem Cells, BMSCs: Bone Marrow Stem Cells

## Transplantation outcomes

Table 2 summarizes the key post-transplant outcome measures for both groups. The results show that the incidence of CMV reactivation was significantly lower in the Early group compared to the Delayed group (*P* = 0.008), while no statistically significant differences were observed for other parameters.

**Table 2:** Clinical outcomes of two groups

Outcomes	Early-HSCT Group	Delayed-HSCT Group	*P* value
N	170	85	
NE engraftment, n, ( % )	165 ( 97.0 )	81 ( 95.2 )	0.346
NE engraftment, d, median ( range )	13 ( 8 ~ 27 )	13 ( 9 ~ 28 )	0.083
PLT engraftment, n, ( % )	143 ( 84.1 )	73 ( 85.8 )	0.773
PLT engraftment, d, median ( range )	16 ( 8 ~ 28 )	16 ( 10 ~ 28 )	0.205
PGF, ( % )	5 ( 2.9 )	4 ( 4.7 )	0.367
SGF, ( % )	4 ( 2.4 )	6 ( 7.1 )	0.079
aGVHD grade, n ( % )	58 ( 34.1 )	32 ( 37.6 )	0.612
II-IV aGVHD grade, n ( % )	22 ( 12.9 )	11 ( 12.9 )	0.987
cGVHD grade, n ( % )	32 ( 18.8 )	23 ( 27.0 )	0.164
Virus reactivation, n ( % )			
CMV	41 ( 24.1 )	34 ( 40.0 )	**0.008**
EBV	102 ( 60.0 )	57 ( 67.1 )	0.213
Response to HSCT			0.067
CR	143 ( 84.1 )	61 ( 71.8 )	
PR	12 ( 7.1 )	9 ( 10.6 )	
NR	15 ( 8.8 )	15 ( 17.6 )	
5-year Survival, n, ( % )	148 ( 87.1 )	68 ( 80.0 )	0.163
5-year GRFS, n, ( % )	126 ( 74.1 )	60 ( 70.5 )	0.585
Death, n, ( % )	22 ( 12.9 )	17 ( 20.0 )	0.131
Infection	10 ( 5.9 )	6 ( 7.1 )	
Hemorrhage	2 ( 1.1 )	2 ( 2.4 )	
Disease progression or relapse	10 ( 5.9 )	9 ( 10.6 )	
Follow-up, m, median ( range )	18 ( 1 ~ 60 )	18 ( 1 ~ 60 )	0.632

Significant P values are in bold type

CR: Complete response. PR: Partial response. NR: No response

**Fig. 1:**
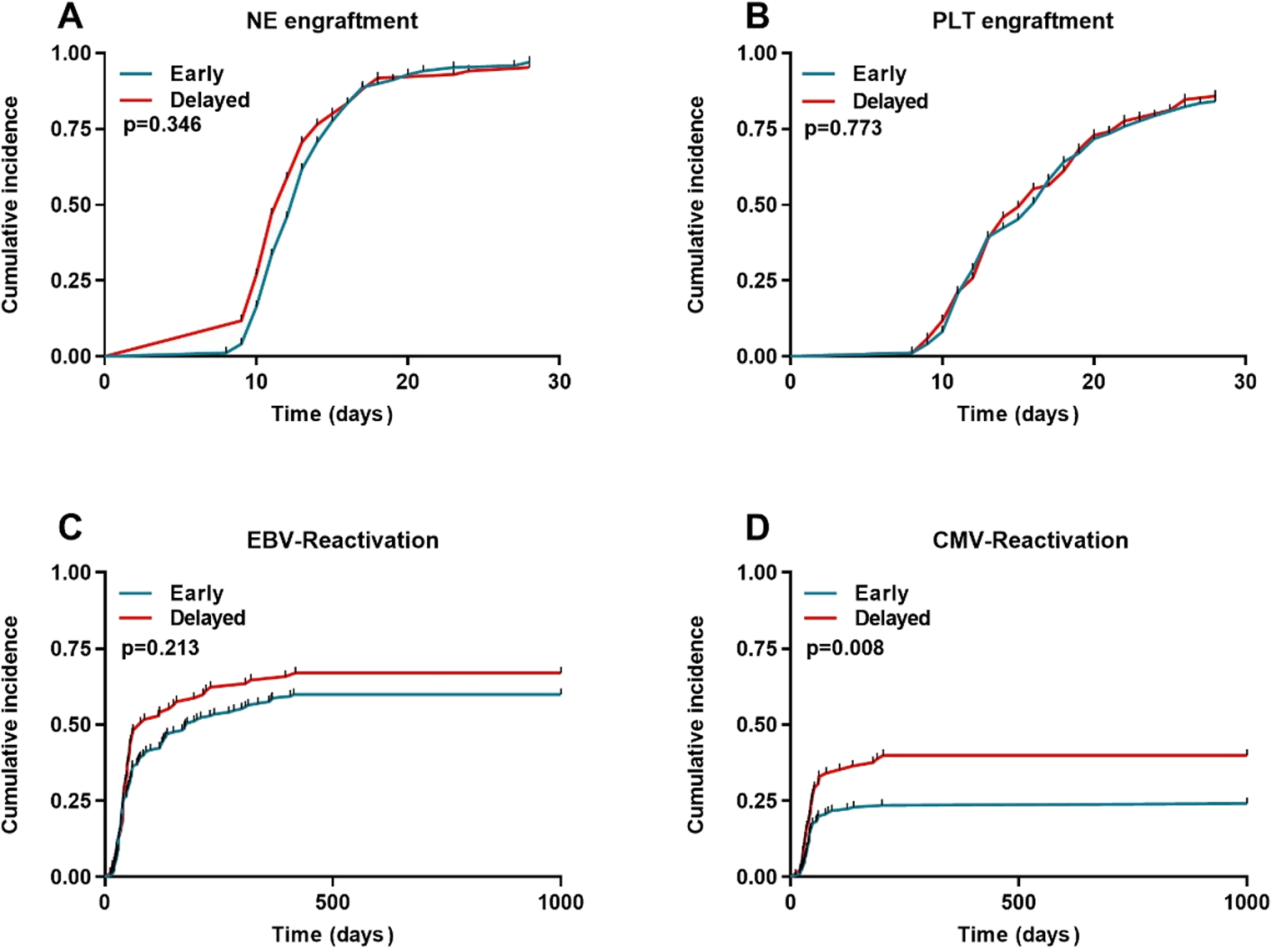
Transplantation outcomes. (**A**) NE engraftment. (**B**) PLT engraftment. (**C**) EBV-reactivation. (**D**) CMV-reactivation

The median number of CD34 + cells in the grafts was 6.01 × 10⁶/kg (range: 1.95 ~ 20.38) for the Early group and 5.99 × 10⁶/kg (range: 1.69 ~ 16.84) for the Delayed group (*P* = 0.193). The cumulative NE engraftment rate was 97.0% in the Early group and 95.2% in the Delayed group (*P* = 0.346) (Fig. 1A). The cumulative PLT engraftment rate was 84.1% in the Early group and 85.8% in the Delayed group (*P* = 0.773) (Fig. 1B). The incidence of primary graft failure was 2.9% (95%CI: 0.6 ~ 5.9%) in the Early group and 4.7% (95%CI: 1.7 ~ 7.3%) in the Delayed group (*P* = 0.367). Corresponding rates of secondary graft failure were 2.4% (95%CI: 0.4 ~ 5.1%) and 7.1% (95%CI: 3.8 ~ 9.7%) (*P* = 0.079), respectively.

The cumulative incidence of EBV reactivation were 60.0% in the Early group and 67.0% in the Delayed group (*P* = 0.213) (Fig. 1C). Unexpectedly, the Early group demonstrated a significantly lower incidence of CMV reactivation compared to the Delayed group (24.1% vs. 40.0%, *P* = 0.008) (Fig. 1D). The incidence of CMV disease was low, at 1.7% and 2.3% in the Early and Delayed groups, respectively (*P* = 0.72).

**Fig. 2:**
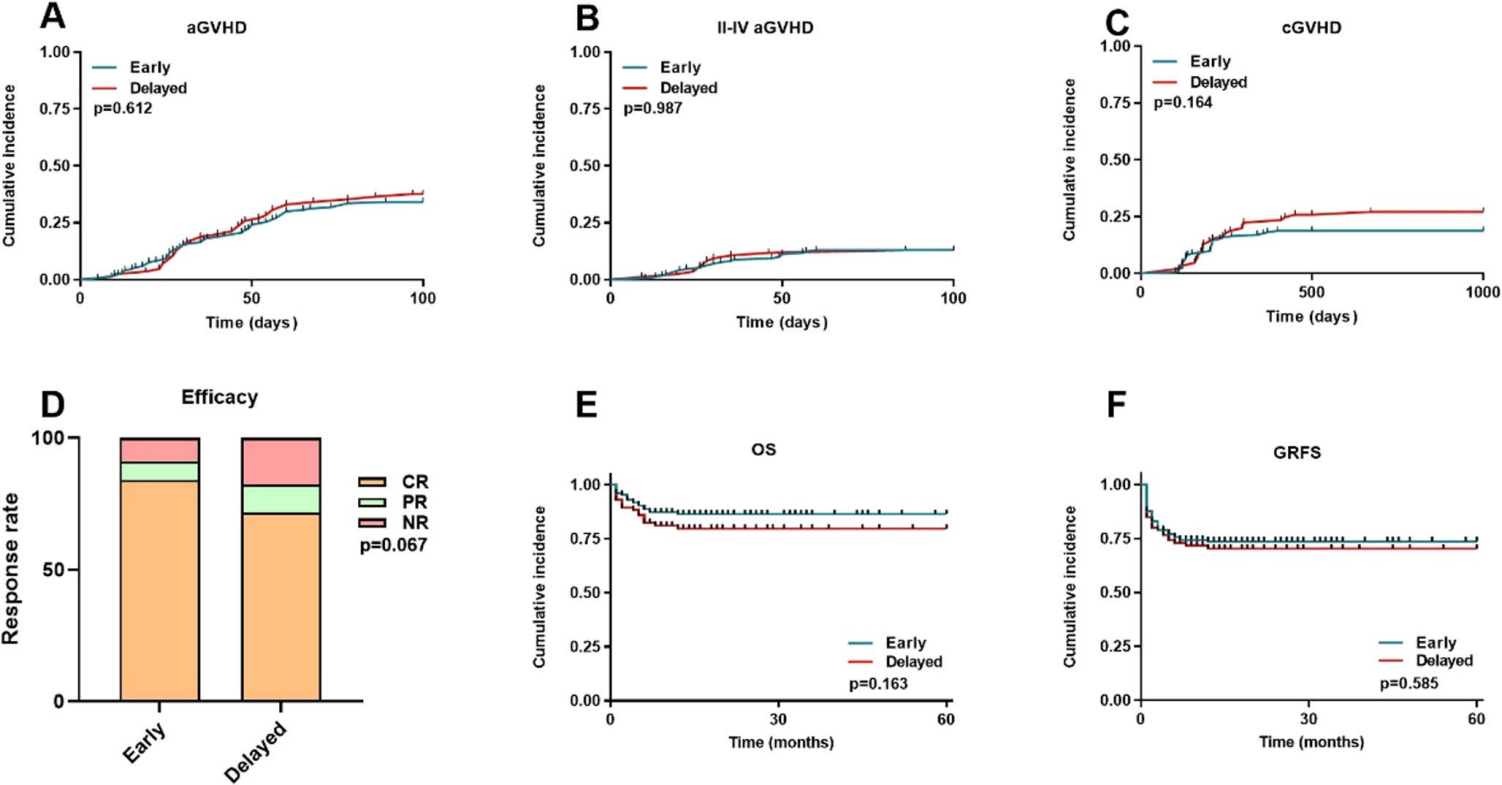
Transplantation outcomes. (**A**) aGVHD. (**B**) Grade II-IV aGVHD. (**C**) cGVHD. (**D**) Efficacy. (**E**) OS. (**F**) GRFS

The cumulative incidence of aGVHD was 34.1% in the Early group and 37.6% in the Delayed group (*P* = 0.612) (Fig. 2A). The cumulative incidence of grade II-IV aGVHD was 12.9% in the Early group and 12.9% in the Delayed group (*P* = 0.987) (Fig. 2B). For cGVHD, the Early group exhibited a relatively lower incidence of 18.8% relative to the Delayed group’s 27.0% (*P* = 0.164) (Fig. 2C). One year post-transplantation, the Early and Delayed groups had OR rates of 91.1% and 82.3% respectively (*P* = 0.067) (Fig. 2D). The 5-year OS was 87.0% in the Early group compared to 80.0% in the Delayed group (*P* = 0.163) (Fig. 2E). The 5-year GRFS was 74.1% in the Early group compared to 70.5% in the Delayed group (*P* = 0.585) (Fig. 2F).

**Fig. 3:**
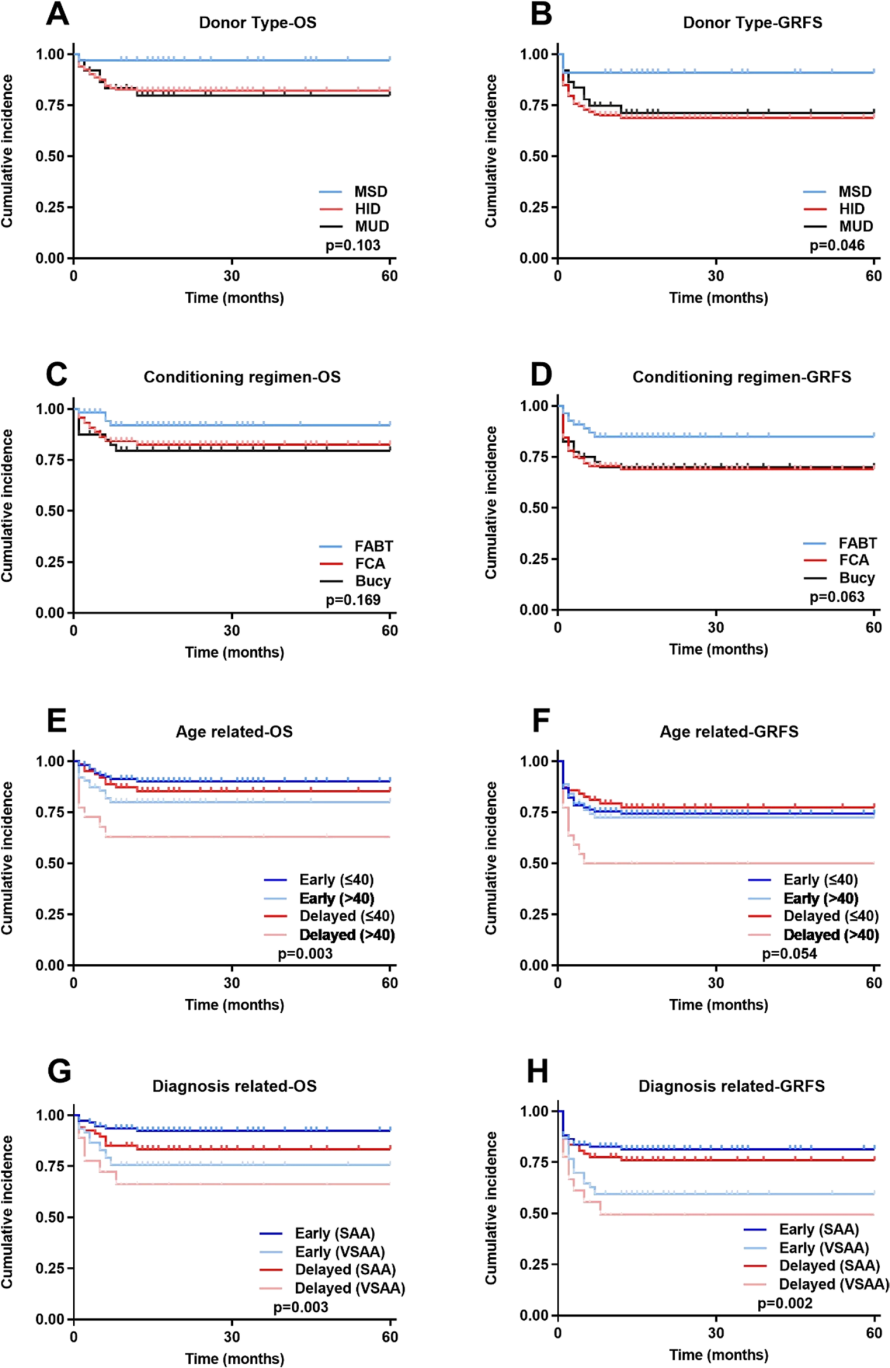
Subgroup analysis. (**A**) Donor Type-OS. (**B**) Donor Type-GRFS. (**C**) Conditioning regimen-OS. (**D**) Conditioning regimen-GRFS. (**E**) Age related-OS. (**F**) Age related-GRFS. (**G**) Diagnosis related-OS. (**H**) Diagnosis related-GRFS

Figure 3 depicts OS and GRFS stratified by donor type, conditioning regimen, patient age, and baseline diagnosis. 5-year OS did not differ significantly among MSD, HID and MUD transplants (*P* = 0.103) (Fig. 1A); however, the MSD cohort exhibited a significantly higher 5-year GRFS than the other two groups (*P* = 0.046) (Fig. 1B). Although no statistically significant differences were observed in 5-year OS (*P* = 0.169) (Fig. 1C) or GRFS (*P* = 0.063) (Fig. 1D) among the three regimens, the survival curves suggest that our novel FABT-based regimen may confer a modest survival advantage and improved quality of life. Patients aged ≤ 40 years achieved superior 5-year OS compared with those > 40 years (*P* = 0.003) (Fig. 1E), whereas the corresponding GRFS demonstrated a favorable trend in the younger cohort that did not reach statistical significance (*P* = 0.054) (Fig. 1F). In the diagnostic subgroup analysis, SAA was associated with better survival than VSAA; moreover, shortening the interval from diagnosis to transplant mitigated the adverse impact of VSAA (Fig. 1G–H).

## Discussion

Allogeneic hematopoietic stem cell transplantation (allo-HSCT) from a matched sibling donor is the only curative treatment for severe aplastic anemia (SAA), particularly in younger patients [8]. Historically, delays in transplantation often resulted from difficulties in donor identification, infection control, and healthcare system barriers. However, over the past decade, the development of haplo-HSCT techniques has alleviated donor shortages and significantly increased transplantation rates [19]. Previous studies have indicated that prolonged intervals from diagnosis to transplantation are associated with increased transfusion dependency, iron overload, infection risk, and clonal evolution, which may adversely affect engraftment and survival [20–23]. According to the European Society for Blood and Marrow Transplantation (EBMT) registry, SAA patients transplanted more than 6 months after diagnosis experienced a 10%~15% reduction in 5-year OS [24]. Nonetheless, much of the existing evidence derives from single-arm or registry-based studies with considerable confounding factors, leaving the optimal timing of transplantation controversial. In this study, we retrospectively analyzed data from 255 SAA patients who underwent first allo-HSCT. Using propensity score matching to control for confounders, we systematically evaluated the impact of the time from diagnosis to transplantation on transplant outcomes. Our results showed no significant differences between the early and delayed groups in key endpoints such as hematopoietic recovery, aGVHD, cGVHD, OS, and GRFS. However, the early group exhibited a significantly lower risk of CMV reactivation and a trend toward improved overall outcomes. These findings suggest that under contemporary transplantation protocols, SAA patients can still achieve favorable survival even when transplantation is performed more than one year after diagnosis, although delayed transplantation may increase the risk of CMV reactivation.

CMV reactivation is a frequent and serious complication following allo-HSCT, which can increase the risk of opportunistic infections, and is strongly associated with the development of GVHD. The elevated risk of CMV reactivation in the delayed group may be mediated by multiple factors. First, long-term transfusion dependence is a typical characteristic of SAA patients. Those in the delayed group received significantly more frequent and larger volumes of blood product transfusions. Since CMV can be transmitted via leukocyte-contaminated blood products, repeated exposure may increase the probability of CMV seroconversion [25]. Second, iron overload can impair the function of macrophages and T cells, thereby weakening the immune surveillance against CMV [26]. Furthermore, patients in the delayed group may experience depletion of lymphocyte subsets due to prior immunosuppressive therapies, particularly functional impairment of CMV-specific T cells. This deficit in immune memory persists during post-transplant immune reconstitution, increasing susceptibility to CMV reactivation [27]. Therefore, for patients with SAA who cannot undergo immediate transplantation for various reasons, enhanced pre-transplant CMV monitoring and consideration of expanding the coverage of anti-CMV prophylactic therapy are warranted.

In contrast to some previous studies, this study did not identify a significant impact of the time interval from diagnosis to transplantation on NE or PLT engraftment. All engraftment rates achieved comparably high levels with no differences. These findings suggest that the impairment of the bone marrow microenvironment in patients with SAA may remain relatively stable between early and advanced disease stages, and that the homing, proliferation, and differentiation capacities of hematopoietic stem cells are not substantially influenced by delayed transplantation. More importantly, modern conditioning regimens effectively ablate recipient hematopoietic tissues and create an immune microenvironment conducive to donor cell engraftment, with efficiency unaffected by transplantation timing. The comparable number of CD34 + cells infused in both groups ensured equivalent “seed” quality, forming a critical basis for the uniformly high engraftment rates. Furthermore, the routine combined use of bone marrow and peripheral blood stem cells as graft sources at our center—where bone marrow components are rich in mesenchymal stem cells and hematopoietic niche-supporting cells [28]—may partially counteract potential adverse microenvironmental alterations associated with delayed transplantation.

GVHD remains a life-threatening complication following haplo-HSCT and a major cause to early mortality, with a markedly higher incidence compared with MSD-HSCT [6, 29, 30]. In the present study, no significant difference in the incidence of GVHD was observed between the two groups. This finding contrasts with reports suggesting that early transplantation reduces GVHD risk, and may instead reflect optimized GVHD prophylaxis strategies employed here. Both groups uniformly received a calcineurin inhibitor combined with MMF, with some patients additionally receiving post-transplant cyclophosphamide (PTCy). This multi-layered immunosuppressive approach effectively controlled GVHD development. However, a trend toward reduced cGVHD was observed in the early transplantation group. Patients undergoing early transplantation, having a shorter disease duration, may possess an immune system that has not fully adapted to aberrant autoreactive states. Under these conditions, establishment of immune tolerance between donor and recipient may be more readily achieved. Furthermore, these patients generally receive immunosuppressive therapy for a shorter period, resulting in a more plastic immune system that is more amenable to the establishment of post-transplant immune homeostasis [31, 32]. In contrast, patients with delayed transplantation often have prolonged exposure to immunosuppressive agents, which may lead to varying degrees of subclinical injury across multiple organ systems. After transplantation, such subclinical damage may promote GVHD onset through the release of damage-associated molecular patterns (DAMPs), which can activate donor T cells [33].

Subgroup analysis identified several key factors influencing prognosis. First, patients aged ≤ 40 years exhibited significantly improved 5-year OS, suggesting that younger patients better tolerate transplantation-related toxicities and possess a distinct survival advantage. Advanced age is associated with declining organ reserve, increased comorbidities, and reduced tolerance to conditioning regimens, collectively contributing to a 31% increase in transplantation-related mortality [34]. In clinical practice, for older patients with SAA, further optimization of conditioning intensity—such as employing reduced-toxicity conditioning regimens or intensifying supportive care—may be necessary to improve outcomes. Second, subgroup analysis by donor type revealed that transplantation from MSD yielded significantly superior 5-year GRFS compared to HID and MUD, which aligns with the current consensus [4]. However, modern HID techniques, through enhanced GVHD prophylaxis and immune modulation, have achieved OS rates comparable to those of MSD transplantation [35]. While HID transplantation addresses donor availability, the HLA barrier necessitates more intensive immunosuppressive therapy, potentially increasing the risk of complications such as infections [36]. For patients lacking an MSD, HID transplantation serves as a preferred alternative, though careful management of cGVHD and its impact on quality of life is warranted. Regarding disease severity, patients with SAA demonstrated better survival than those with VSAA, and early transplantation mitigated the adverse prognostic impact of VSAA. VSAA patients typically present with more profound baseline hematopoietic failure, higher risks of infection, and often experience a more pronounced inflammatory state prior to transplantation, all of which increase the complexity of the procedure [37]. Early transplantation, by shortening the duration of severe cytopenias, reduces infection- and transfusion-related complications, thereby improving outcomes in VSAA patients. In terms of conditioning regimens, the FABT regimen—utilizing reduced-dose busulfan and thiotepa, combined with low-dose ATG and PTCy for GVHD prophylaxis—was designed to balance myeloablation and toxicity control. Survival curves indicated a trend toward superiority over FCA and BuCy regimens.

Most current guidelines recommend transplantation as first-line treatment for eligible young AA patients with suitable donors [14]. The majority of centers in China have accumulated extensive experience in proceeding directly to transplantation upon diagnosis, especially in haplo-HSCT [38, 39]. In this cohort, only 2.4% of patients in the Early group and 5.9% in the Delayed group had received prior IST, demonstrating that the vast majority underwent transplantation without an IST history. This approach offers several distinct advantages: First, it eliminates the risks of severe infection, hemorrhage, and clonal evolution during periods of ineffective IST [40]; second, it avoids prolonged immune suppression from IST agents, thereby preserving optimal T-cell functional status; and third, it streamlines the treatment algorithm while reducing overall waiting time. Our data demonstrate that HID donors accounted for over 70% of the study population, confirming that HID-HSCT has become the predominant choice for Chinese SAA patients. The high donor availability and short waiting times inherent to this approach enable transplantation timing to be tailored to patient condition rather than being constrained by donor search logistics. This “first-line transplantation” strategy not only ensures therapeutic efficacy but also substantially reduces overall healthcare burden, offering a valuable reference for global AA management.

In summary, this study demonstrates that within the framework of contemporary allo-HSCT, early transplantation significantly reduces the risk of CMV reactivation and improves clinical outcomes in patients with newly diagnosed SAA, particularly VSAA. Therefore, we recommend expediting pre-transplantation evaluation and donor screening processes. For elderly patients or those with significant comorbidities, the preparatory period may be reasonably extended to optimize transplantation conditions. This study has several limitations. First, despite the application of PSM, residual confounding factors may still affect the results. Second, the single-center design restricts the generalizability of our findings, as inter-center heterogeneity in conditioning regimens, GVHD prophylaxis strategies, CMV monitoring protocols, and preemptive intervention thresholds may limit the extrapolation of our conclusions. Additionally, donor type may exert a potential influence on transplantation timing. While historically a factor affecting waiting times, China’s large population base and highly efficient donor screening system have substantially narrowed actual waiting time differences across donor types. Future research should focus on conducting prospective randomized controlled trials to validate the clinical benefits of early transplantation and to further elucidate the molecular mechanisms by which early intervention influences prognosis. These initiatives are aimed at formulating more precise and effective treatment strategies for SAA patients, with the ultimate goal of enhancing their long-term quality of life.

## Data Availability

The data that support the findings of this study are available from the corresponding author, [Wenbin Liu or Baodong Ye], upon reasonable request.
